# Induction of High Levels of Specific Humoral and Cellular Responses to SARS-CoV-2 After the Administration of Covid-19 mRNA Vaccines Requires Several Days

**DOI:** 10.3389/fimmu.2021.726960

**Published:** 2021-10-04

**Authors:** Sergio Gil-Manso, Diego Carbonell, Luis López-Fernández, Iria Miguens, Roberto Alonso, Ismael Buño, Patricia Muñoz, Jordi Ochando, Marjorie Pion, Rafael Correa-Rocha

**Affiliations:** ^1^ Laboratory of Immune-Regulation, University Hospital Gregorio Marañón and “Gregorio Marañón” Health Research Institute (IISGM), Madrid, Spain; ^2^ Department of Hematology, University Hospital Gregorio Marañón and “Gregorio Marañón” Health Research Institute (IISGM), Madrid, Spain; ^3^ Pharmacy Service, University Hospital Gregorio Marañón and “Gregorio Marañón” Health Research Institute (IISGM), Madrid, Spain; ^4^ Emergency Service, University Hospital Gregorio Marañón and “Gregorio Marañón” Health Research Institute (IISGM), Madrid, Spain; ^5^ Department of Clinical Microbiology and Infectious Diseases of the University Hospital Gregorio Marañón and “Gregorio Marañón” Health Research Institute (IISGM), Madrid, Spain; ^6^ School of Medicine, Complutense University of Madrid, Madrid, Spain; ^7^ Precision Immunology Institute, Icahn School of Medicine at Mount Sinai, New York, NY, United States; ^8^ Centro Nacional de Microbiología, Instituto de Salud Carlos III, Madrid, Spain

**Keywords:** COVID-19, mRNA-vaccines, specific humoral response, specific T-cell response, SARS – CoV – 2

## Abstract

**Objectives:**

In the context of the Covid-19 pandemic, the fast development of vaccines with efficacy of around 95% preventing Covid-19 illness provides a unique opportunity to reduce the mortality associated with the pandemic. However, in the absence of efficacious prophylactic medications and few treatments for this infection, the induction of a fast and robust protective immunity is required for effective disease control, not only to prevent the disease but also the infection and shedding/transmission. The objective of our study was to analyze the level of specific humoral and cellular T-cell responses against the spike protein of SARS-CoV-2 induced by two mRNA-based vaccines (BNT162b2 and mRNA-1273), but also how long it takes after vaccination to induce these protective humoral and cellular immune responses.

**Methods:**

We studied in 40 healthy (not previously infected) volunteers vaccinated with BNT162b2 or mRNA-1273 vaccines the presence of spike-specific IgG antibodies and SARS-CoV-2-specific T cells at 3, 7 and 14 days after receiving the second dose of the vaccine. The specific T-cell response was analyzed stimulating fresh whole blood from vaccinated volunteers with SARS-CoV-2 peptides and measuring the release of cytokines secreted by T cells in response to SARS-CoV-2 stimulation.

**Results:**

Our results indicate that the immunization capacity of both vaccines is comparable. However, although both BNT162b2 and mRNA-1273 vaccines can induce early B-cell and T-cell responses, these vaccine-mediated immune responses do not reach their maximum values until 14 days after completing the vaccination schedule.

**Conclusion:**

This refractory period in the induction of specific immunity observed after completing the vaccination could constitute a window of higher infection risk, which could explain some emerging cases of SARS-CoV-2 infection in vaccinated people.

## Introduction

The current Covid-19 pandemic caused by severe acute respiratory syndrome coronavirus 2 (SARS-CoV-2) has caused more than 3 million deaths and enormous economic and social upheaval internationally. An unprecedented research effort has resulted in the fast development of Covid-19 vaccines in less than one year, and more than 80 vaccine candidates are in clinical development at present (1, 2). Two of the vaccines developed, BNT162b2 (Pfizer-BioNTech) and mRNA-1273 (Moderna), are based on encapsulated mRNA encoding as the target antigen the spike (S) glycoprotein of the virus, and they are being massively administered around the world. Initial clinical trials employing these two vaccines report efficacy of around 95% preventing Covid-19 illness. In the case of BNT162b2, the primary endpoint was the efficacy of the vaccine against Covid-19 with onset at least 7 days after the second dose (3). Regarding mRNA-1273 vaccine, the primary endpoint was the vaccine’s efficacy in preventing the first occurrence of symptomatic Covid-19 with onset at least 14 days after the second injection (4).

However, in the absence of efficacious prophylactic medications and few treatments for this infection, effective disease control requires a vaccine capable of reducing not only the disease but also the infection and shedding/transmission. Comprehensive studies about the degree and time course of the immunization induced by these vaccines could provide relevant information to resolve some critical questions: how many days are required to generate a protective barrier against the infection after the vaccine administration? Which degree of both humoral and cellular immunity against SARS-CoV-2 are induced by the vaccine? Is the induced immunity capable of clearing the virus? Recent studies confirm that administration of these vaccines elicits neutralizing antibodies against the virus (5, 6). However, less is known regarding the vaccine-mediated induction of cellular responses of adaptive immunity, which are crucial in controlling the virus and notably in acquiring an immunizing memory against the virus (7).

In this study, we analyzed the short-term induction of specific humoral and cellular T-cell responses against the spike protein of SARS-CoV-2 at 3, 7 and 14 days after completing the entire vaccination schedule of the two main mRNA-based vaccines that are being administered massively in the world.

## Methods

### Cohort Description

We performed a prospective observational study in 40 healthy naïve (not previously infected) volunteers distributed in two groups, individuals vaccinated with BNT162b2 (n=21) or vaccinated with mRNA-1273 (n=19). The study was conducted after the approval of the University Hospital Gregorio Marañón ethics committee. Informed written consents from the volunteers were obtained before enrolment. Volunteers were hospital workers from University Hospital Gregorio Marañón of Madrid (Spain), who received the second vaccine dose between January and February 2021. Mean age (± standard error of the mean (SEM)) was 41.05 (± 2.80) for volunteers receiving BNT162b2 and 38.11 (± 2.13) for volunteers receiving the mRNA-1273 vaccine. A description of demographical and clinical characteristics is shown in **Table 1**. There were no significant differences for these variables between the volunteers receiving the BNT162b2 or the mRNA-1273 (p>0.05).

**Table 1:** Demographic and clinical characteristics of the BNT162b2 and mRNA-1273 volunteers.

Characteristics	BNT162b2	mRNA-1273	*p*-value
**Number of Patients**	21	19	
**Age** (years), **median** (SEM)	41.05 (±2.80)	38.11 (±2.13)	0.416
**Gender**			0.711
Male	4	5	
Female	17	14	
**Ethnicity**			–
Caucasian	21	19	
**Comorbidities**			
Current smoker	5	4	1
Ex-smoker	3	0	0.232
Hypertension	1	0	1
Heart disease	0	0	–
Immune disease	1	0	1
Obesity	0	1	0.475
Diabetes	0	1	0.475
Pulmonary disease	4	0	0.107
Renal disease	0	0	–
Hepatic disease	0	0	–
Neurological disease	1	0	1
**Treatment**			
Acetaminophen	18	12	0.148
Ibuprofen	2	4	0.397

Volunteers were divided into BNT162b2 or mRNA-1273 groups according to the vaccine received. Two-sample t-test with equal variances was used for comparison of age, and chi-squared tests were used for the rest of the demographic variables of the volunteers. p-value < 0.05 indicated statistical difference between groups. SEM, standard error of the mean.

### Methodology

Peripheral blood samples were collected on days 3, 7 and 14 after administering the second dose and completing the vaccination schedule at the General University Hospital Gregorio Marañón.

We measured in whole blood IgG specific antibodies for the spike protein (S) and the nucleocapsid (N) at different time points (3-, 7- and 14-days post-vaccination). For that, we employed the *Architect SARS-COV-2 anti-N IgG* and *Architect SARS-COV-2 anti-S IgG* reagents on an *Architech* autoanalyzer for immunoassay based on magnetic microparticle capture technology and chemiluminescence detection (CLIA), all from Abbott diagnostics.

We also quantified SARS-CoV-2-specific T cells employing a direct and quantitative *ex vivo* measurement of IFN-γ, IL-2, IL-4 and IL-10 release by whole blood samples stimulated with SARS-CoV-2 peptides, as previously described (8). Briefly, freshly extracted whole blood was directly stimulated with peptides pools derived from SARS-CoV-2 (*PepTivator*, Miltenyi Biotech). For the stimulation, we mixed spike(S)-peptides *PepTivator SARS-CoV-2 Prot S, Prot S+* and *Prot S1* (Miltenyi Biotech), generating the “pool S”, which cover the whole sequence of the spike. We also mixed nucleocapsid (N) and membrane (M) peptides *PepTivator SARS-CoV-2 Prot N* and *Prot M*, generating the “pool NM”, used as a control. These two pools consisted mainly of 15-mer sequences overlapping the complete sequence of spike, membrane and nucleocapsid of the SARS-CoV-2 sequences (GenBank MN908947.3). We stimulated whole blood samples with pool S and pool NM at 1 µg/ml (according to manufacturer’s instructions), incubating 14 – 16 hours at 37°C and 5% CO_2_. After incubation, plasma from stimulated samples was recovered and stocked at -80°C until its analysis. Plasma was analyzed using the microfluidic ELISA equipment *ELLA-Protein Simple* (Biotechne), measuring the concentration of several cytokines released after stimulation (IFN-γ, IL-2, IL-4 and IL-10).

Statistical analyses were conducted at the IISGM biostatistics facility. A chi-squared test was used for categorical variables and a t-test for quantitative variables to compare demographic and clinical variables. In all other analyses, variables were log-transformed to obtain normal distributions and increase statistical power. The evolution over time of the different variables was studied using linear mixed-effects regression model, considering each individual as a random effect and the vaccination as a fixed effect with an unstructured covariance structure. These models have great flexibility and power to study variables repeatedly measured over time with missing values (8). Graphics were made with *GraphPad Prism* software, and statistical analysis was performed with *Stata* software. All statistical tests were 2-sided, and a P-value of p<0.05 was considered statistically significant.

## Results

### Cohort Description

Mean age (± standard error of the mean (SEM)) was 41.05 (±2.80) for volunteers receiving BNT162b2 and 38.11 (±2.13) for volunteers receiving mRNA-1273 vaccine. 81% of volunteers were women in the BNT162b2 group, and 73.7% in the mRNA-1273. There were no significative differences in age, sex, and other standard demographic variables (**Table 1**) between the two groups of individuals (p>0.05).

### Specific Humoral Response

**Figure 1:**
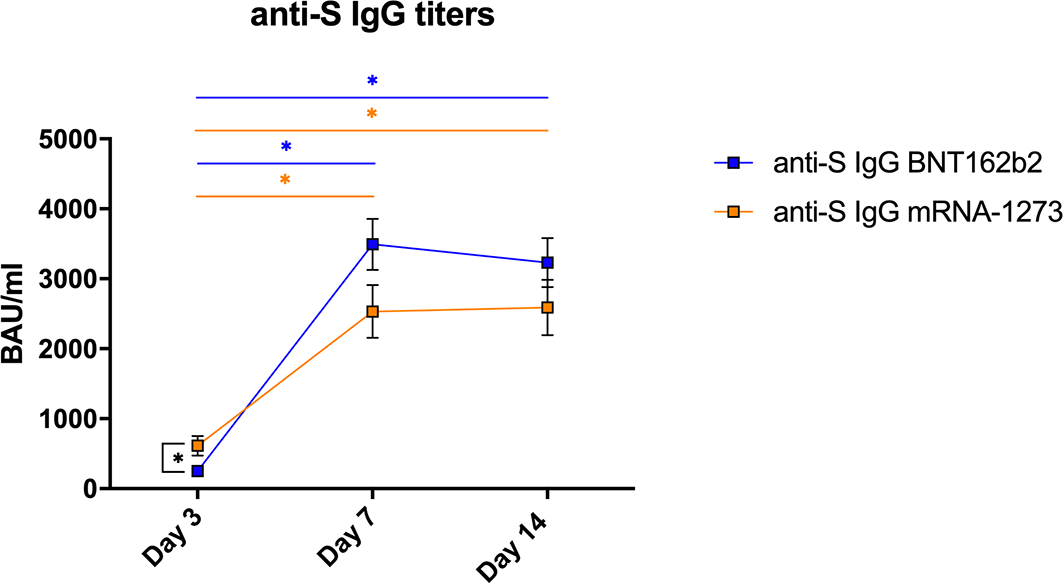
Specific humoral responses in vaccinated healthy subjects. Mean±SEM values for S-protein specific IgG antibodies (BAU/ml) measured in plasma from volunteers vaccinated with BNT162b2 (n=21) or mRNA-1273 (n=19) after 3, 7 and 14 days of receiving the second dose of the vaccine. The linear mixed-effects regression model was adjusted to evaluate each immunological covariate’s evolution over time in each group. *Significant difference when p < 0.05.

All vaccinated subjects enrolled in the study showed a clear humoral response to the vaccine reaching high titers of anti-S IgG (>800 WHO binding antibodies units (BAU)/ml). However, there was a delay in the response after completing the vaccination, and specific IgG were significantly lower at day 3 post-vaccination than at day 7 or 14 in both groups (p<0.001; **Figure 1**).

We compared the capacity between vaccines to induce a specific humoral IgG response. Employing a linear mixed-effects regression model, the analysis indicated that the evolution on this response was different for each vaccine (p<0.001). Patients vaccinated with mRNA-1273 had higher IgG-S titers at day 3 (p=0.003) (mean±SEM BNT162b2= 250.8±31.8; mRNA-1273= 613.1±138.2 BAU/ml), but the values reached at day 7 (BNT162b2= 3491.7±366.3; mRNA-1273= 2532.6±378.3 BAU/ml) or day 14 (BNT162b2= 3229.3±349.7; mRNA-1273= 2589.7±396.4 BAU/ml) were comparable with both vaccines (**Figure 1**).

### Specific T-Cell Response

**Figure 2:**
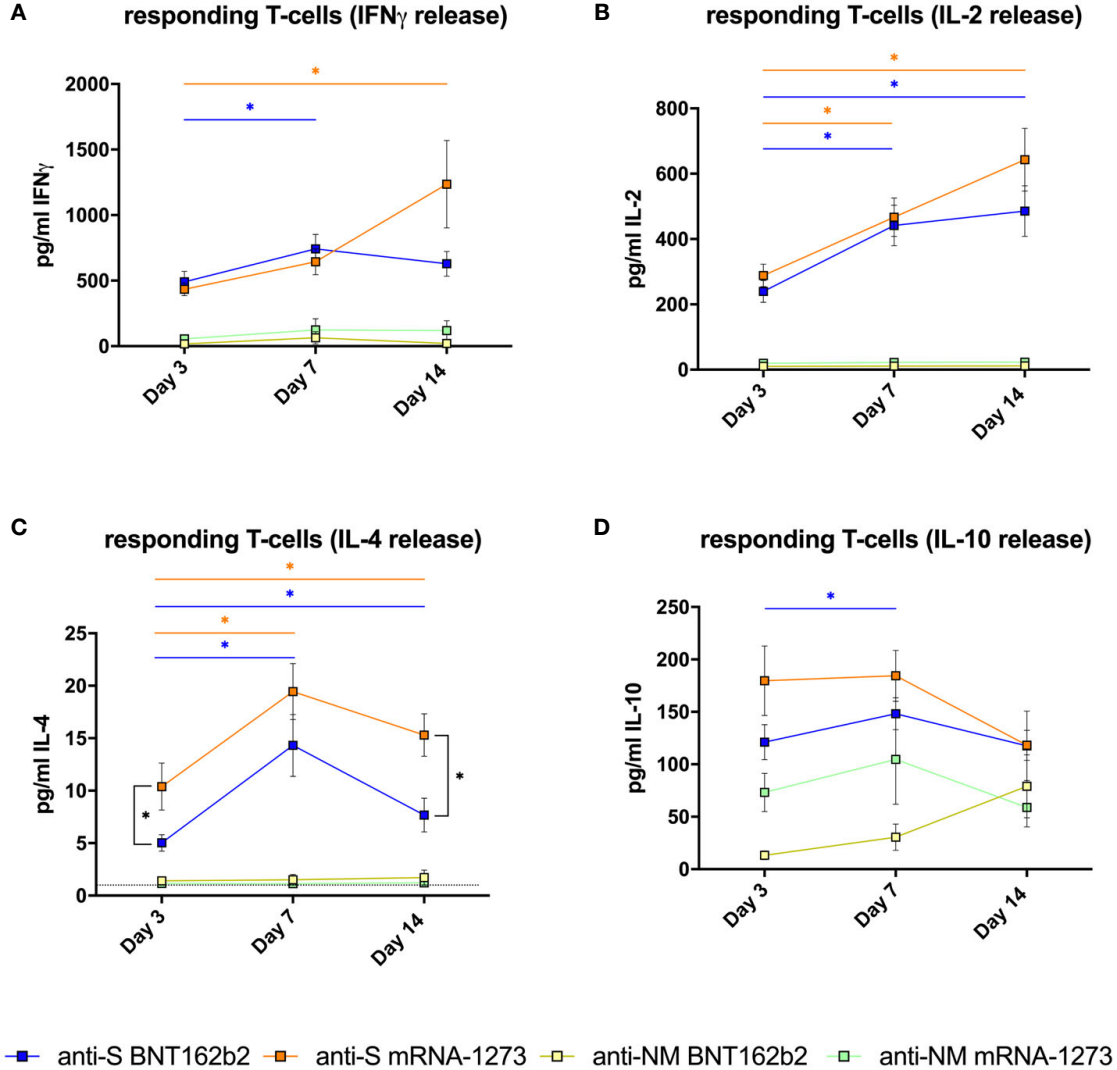
Specific T-cell responses in vaccinated healthy subjects. **(A)** Mean±SEM T-cell responses measured by IFNγ release (pg/ml); **(B)** IL-2 release (pg/ml); **(C)** IL-4 release (pg/ml) and **(D)** IL-10 release (pg/ml) **(D)** after *in vitro* stimulation with spike-derived peptides pool (S) or mixed nucleocapsid and membrane peptides (NM) from volunteers vaccinated with BNT162 (n=21) or mRNA-1273 (n=19) after 3, 7 and 14 days of receiving the second dose of the vaccine. The dotted line in **(C)** indicates the lower detection limit of the method for this cytokine, showing that the low concentrations detected are reliable. The linear mixed model was adjusted to evaluate each immunological covariate’s evolution over time in each group. *Significant difference when p < 0.05.

The presence of **responding T cells** to the specific stimulation with SARS-CoV-2 peptides was measured as the cytokine release after stimulation with specific peptides. High values (> 400 pg/mL) of IFN-γ, which is a key cytokine in the antiviral Th1 response, were observed as early as day 3 after completing vaccination only in samples stimulated with S peptides, and the magnitude was comparable for both vaccines (p=0.874). The linear mixed-effects regression model indicates that the evolution of the frequency of these specific T cells was different between the two vaccines (p=0.017). The response seems to increase faster in patients vaccinated with BNT162b2 reaching the maximal values at day 7 (BNT162b2= 742.1±110.8; mRNA-1273= 593.4±101.6, pg/ml±SEM) and maintaining these values at day 14. However, patients receiving mRNA-1273 reached higher maximal values at day 14 (BNT162b2= 628.7±94.6; mRNA-1273= 1231.4±333.8) (**Figure 2A**).

Similar behavior was observed in the analysis of IL-2 release (**Figure 2B**). High values (> 200 pg/ml IL-2) were observed at day 3 after the second dose (BNT162b2= 239.6±33.6; mRNA-1273= 288.6±34.3, pg/ml±SEM). Comparing the two vaccines, values of IL-2 release were comparable in both groups at 3, 7 and 14 days (p>0.05), and the evolution on time of IL-2 release capacity was also similar with both vaccines (p=0.319). The IL-2 release increased significantly (p<0.05) in both groups from day 3 to day 7 (BNT162b2= 441.7±61.8; mRNA-1273= 466.9±58.7, pg/ml±SEM), and continue to increase progressively until reaching the maximal values at day 14 (BNT162b2= 485.5±77.4; mRNA-1273= 642.9±96.2, pg/ml±SEM) (**Figure 2B**), which were not significantly different between the two vaccines.

Regarding IL-4 release, which is a cytokine related to Th-2 responses, we observed a significant increase from day 3 to day 7 with both vaccines (p<0.001), and then a slight decrease was observed from day 7 to day 14 (**Figure 2C**). The evolution on time of IL-4 producing T cells in the two groups was comparable (p=0.165). However, the values of IL-4 release reached at day 3 (p=0.035) and day 14 (p<0.001) were significantly higher in subjects vaccinated with mRNA-1273 vaccine than with BNT162b2.

The two mentioned vaccines codify for protein-S mRNA, and therefore should only induce immunity against the S protein of the virus. The absence of both IgG against nucleocapsid and T-cell response (IFN-γ, IL-2 and IL-4 release) after stimulation with SARS-Cov-2 peptides from nucleocapsid and membrane (NM), which was comparable to the non-stimulated condition (data not shown), indicate that the observed response is a direct consequence of the vaccination and is not due to a previous exposition to the virus or asymptomatic infection.

Regarding IL-10 (which at difference of IFN-γ, IL-2 and IL-4 is an anti-inflammatory cytokine), the evolution on time of IL-10 release after stimulation with S peptides was similar in both groups (p=0.838), and IL-10 values at day 3, 7 and 14 were comparable with both vaccines (p>0.05) (**Figure 2D**). We detected some IL-10 release in the samples stimulated with NM peptides, although the values in samples stimulated with S peptides were clearly higher (p<0.001). However, independently of the peptide employed, the IL-10 release in stimulated samples was higher than in non-stimulated samples.

## Discussion

Several recent articles describe that mRNA-based vaccines elicit neutralizing antibodies that persist for several months. In the case of the mRNA-1273 vaccine, mRNA1273-elicited binding and neutralizing antibodies were present in vaccinated healthy adults 180 days (6 months) after the second dose (5). A pre-printed work (not yet peer-reviewed) also indicates that a single dose of the BNT162b2 vaccine elicits antibodies. However, there are no data about the persistence of neutralizing antibodies in individuals receiving this vaccine (6). On the other hand, there is little information about the degree of specific T-cell responses induced by the vaccination and also how long it takes after vaccination to induce protective humoral and cellular immune responses.

In our study, there is a higher proportion of females in both groups. It has been described that women could be more resistant to SARS-CoV-2 infection compared to males. However, in the case of the mRNA vaccines, it seems that gender is not implicated in their effectiveness since both males and females present similar protection against symptomatic infection after vaccination (9). Therefore, the effectiveness, which would be related to the degree of vaccine-induced immune responses, seems to be independent of sex. Since no difference was seen in the frequency of women between both groups, the comparison of the immune response induced by the two vaccines would not be influenced by the differences in the female proportion of our cohort.

The values of specific antibodies or T cells required to confer a protective immunity capable of preventing infection are unknown. Some studies suggest that a neutralizing titer equivalent to 20% of the average convalescent titer is sufficient to provide 50% protection from symptomatic Covid-19. Still, it is unknown the levels of specific antibodies to block viral entry and replication (10). Regarding the adaptive immunity mediated by T cells, spike-specific memory CD4+ T cells are detected in most convalescent individuals after 6 months post-infection (11). However, less is known about the influence of the level of specific T-cell immunity in the clinical progression and viral clearance.

Awaiting further studies to clarify the level of immunity required to confer protection, our results indicate that a delay after completing the vaccination schedule to reach high levels of anti-S IgG antibodies could exist. Even if serum IgG takes more time to be elicited than IgM after vaccination, a recent article shows that IgG could be detected after the first dose of the anti-SARS-CoV-2 vaccine (12). However, in the light of our results, the second dose could be essential to maintain the sustained production of anti-SARS-CoV-2 immunoglobulins. As we showed in this manuscript, IgG anti-SARS-CoV-2 are already present 3 days after the second dose. However, the maximal level of protection seemed to be established only 7 days after, showing that the second dose might be pivotal for strong protection. The reduced levels of specific antibodies in this period could be determinant in the capacity of vaccinated people to block initial infection, control viral replication, and consequently, in the clinical progression and capacity of transmission (10).

Regarding cellular T-cell responses, both vaccines demonstrate to induce a fast specific T-cell response, and the presence of anti-S responding T cells secreting IFN-γ, IL-2 or IL-4 was remarkable as soon as day 3 post-vaccination. IFN-γ is a crucial cytokine produced notably by T helper 1 (Th1) CD4 T cells, which are the main immune mediators in the control of intracellular pathogens such as viruses. However, the maximal levels of IFN-γ and IL-2 release, were reached at day 7 (BNT162b2) or day 14 (mRNA-1273) post-vaccination. We also quantified IL-2, a cytokine related to the proliferation and activation of the whole immune cells, and we observed maximum values at day 14 for both vaccines, indicating that specific T-cells responded to derived-peptides releasing IL-2 too.

The stimulation with SARS-CoV-2 peptides also revealed the presence of specific T-cells able to produce IL-4, which is a Th2-related cytokine. The presence of these specific IL4-secreting cells seems to be a consequence of vaccination because there was no liberation of IL-4 in the presence of NM peptides. Polarization of CD4 T cells to a Th2 profile might potentially be detrimental, as they have been linked to vaccine-associated enhanced respiratory disease (13). However, IL-4 secreted by T-follicular helper (Tfh) cells, which are crucial regulators of germinal centres and affinity-matured Ab responses, could also provide a beneficial effect as it mediates an anti-apoptotic helper function on B cells (14). The highest plasma-neutralizing activity in patients recovered from COVID-19 has been associated with increased frequencies of Th1- and Th2-biased circulating Tfh cells (15).

In agreement with our results, Bettini et al. describe in a recent review that immunization in animal models with SARS-CoV-2 mRNA-vaccines elicited a robust production of Th1 cytokines (including IFN-γ, TNF, and IL-2). In addition, the presence of Tfh characterized by the production of both Th1 (IFN-γ) and Th2 (IL-4) cytokines was also observed in mice immunized with these vaccines (14). Therefore, because we are analyzing cytokines released by total T cells, we are probably observing the presence of both specific Th1 and Tfh cells, which will constitute a desirable effect of the vaccination. We hypothesize that the decrease in IL-4 release observed between day 7 and day 14 could also reflect a higher polarization to a Th1 (IFN-γ) response at this time, which in the case of patients receiving the mRNA-1273 vaccine would coincide with reaching the maximum levels of IFN release. In the light of our results, the mRNA-1273 vaccine seems to produce a higher increase in the cells producing this IL-4, but because the concentration of this cytokine is still reduced (< 20 pg/ml), we cannot conclude whether this difference will be relevant to the physiological level.

Interestingly, we also observed high values of IL-10 release in all the stimulated conditions independently of the peptides employed. IL-10 is a suppressive cytokine mainly produced by Treg cells in response to immune activation (16). The increase of IL-10 after stimulation with NM-peptides could be related to a response from Tregs to the processing and presentation of peptides by antigen-presenting cells, even in the absence of a specific T-cell response. Another potential explanation for this increase in IL-10 release could be an unspecific activation of Tregs during the incubation *in vitro* of blood samples with peptides. The fact that IL-10 release is much higher in the presence of S peptides than with NM is probably related to a response from Treg to the activation of S-specific T cells in vaccinated subjects and the subsequent increase observed in the production of IL-2, which is the main factor to active and to increase the proliferation of Tregs. Moreover, the presence of IL-2 has been shown to specifically enhance the production of IL-10 by human Tregs (17), which would explain a higher IL-10 release in the presence of S peptides.

In conclusion, the short-term induction of specific humoral and cellular responses was comparable for both vaccines, with slight differences in magnitude and the time required to reach the maximal responses. In both groups, the values of specific humoral and cellular immunity were significantly lower at day 3 than at day 7 or 14 post-vaccination, indicating the presence of a potential refractory period to reach the maximal response. This refractory period in the induction of specific immunity observed after completing the vaccination could constitute a window of higher infection risk, which could explain some emerging cases of SARS-CoV-2 infection after the first dose of the vaccine (18) or even among subjects with the complete vaccination schedule (19).

Further studies are required to elucidate the minimal values for both IgG titers and T-cell response necessary to confer a protective immunization and to know the persistence of these protective immunizations. This information will be crucial to evaluate the degree of effective immunization in the population and whether the administration of booster vaccine doses is required if immune responses are lost after several months.

## Data Availability Statement

The raw data supporting the conclusions of this article will be made available by the authors, without undue reservation.

## Ethics Statement

The study was conducted after the approval of the University Hospital Gregorio Marañón ethics committee. The patients/participants provided their written informed consent to participate in this study.

## Author Contributions

SG-M and DC performed experiments and analyzed data. LL-F, IB, and IM participated in the recruitment of volunteers, collection of samples and the patients’ data. RA and PM analyzed antibody titers. JO developed the protocol of T-cell analysis and contributed to the design of the study. All authors interpreted and discussed the data. RC-R and MP designed the study, interpreted the results, wrote and revised the manuscript. All authors contributed to the article and approved the submitted version.

## Funding

This work was supported by grants co-funded by ERDF (FEDER) Funds from the European Commission “A way of making Europe” from the Instituto de Salud Carlos III (ISCIII) COV20-00668 to RCR and PI18/00506 to MP. It was also partially financed by a grant from “Fundación Familia Alonso” (FFA-FIBHGM-2019) and the consortium ACT4COVID, funded by Cellnex-Telecom. SG-M was supported by the Youth Employment Program co-financed by the Madrid community and FEDER Funds (PEJ-2020-AI/BMD-17954). JO has received funding from the European Union Horizon 2020 research and innovation program VACCELERATE under grant agreement No [101037867]. The funders had no role in study design, data collection, or analysis; the decision to publish; or the preparation of the manuscript.

## Conflict of Interest

The authors declare that the research was conducted in the absence of any commercial or financial relationships that could be construed as a potential conflict of interest.

## Publisher’s Note

All claims expressed in this article are solely those of the authors and do not necessarily represent those of their affiliated organizations, or those of the publisher, the editors and the reviewers. Any product that may be evaluated in this article, or claim that may be made by its manufacturer, is not guaranteed or endorsed by the publisher.
